# Development of Machine Learning-Based Web System for Estimating Pleural Effusion Using Multi-Frequency Bioelectrical Impedance Analyses

**DOI:** 10.3390/jcdd10070291

**Published:** 2023-07-07

**Authors:** Daisuke Nose, Tomokazu Matsui, Takuya Otsuka, Yuki Matsuda, Tadaaki Arimura, Keiichi Yasumoto, Masahiro Sugimoto, Shin-Ichiro Miura

**Affiliations:** 1 Department of Cardiology, Fukuoka University Faculty of Medicine, Fukuoka 814-0180, Japan; kirinnwine@yahoo.co.jp; 2 Department of Cardiology, Fukuoka Heartnet Hospital, Fukuoka 819-0002, Japan; 3 Research Institute for Advanced Medical Development for Heart Failure, Fukuoka University, Fukuoka 814-0180, Japan; 4 Graduate School of Science and Technology, Nara Institute of Science and Technology, Nara 690-0101, Japan; m.tomokazu@is.naist.jp (T.M.); yukimat@is.naist.jp (Y.M.); yasumoto@is.naist.jp (K.Y.); 5 Technical Sales Department, Dialysis Division, Toray Medical Company Limited, Tokyo 103-0023, Japan; takuya.ootsuka.g6@mail.toray; 6 Institute for Advanced Biosciences, Keio University, Tsuruoka 997-0035, Japan; mshrsgmt@tokyo-med.ac.jp; 7 Institute of Medical Science, Tokyo Medical University, Tokyo 160-0023, Japan

**Keywords:** heart failure, impedance, device, estimation system, machine learning

## Abstract

Background: Transthoracic impedance values have not been widely used to measure extravascular pulmonary water content due to accuracy and complexity concerns. Our aim was to develop a foundational model for a novel system aiming to non-invasively estimate the intrathoracic condition of heart failure patients. Methods: We employed multi-frequency bioelectrical impedance analysis to simultaneously measure multiple frequencies, collecting electrical, physical, and hematological data from 63 hospitalized heart failure patients and 82 healthy volunteers. Measurements were taken upon admission and after treatment, and longitudinal analysis was conducted. Results: Using a light gradient boosting machine, and a decision tree-based machine learning method, we developed an intrathoracic estimation model based on electrical measurements and clinical findings. Out of the 286 features collected, the model utilized 16 features. Notably, the developed model demonstrated high accuracy in discriminating patients with pleural effusion, achieving an area under the receiver characteristic curves (AUC) of 0.905 (95% CI: 0.870–0.940, *p* < 0.0001) in the cross-validation test. The accuracy significantly outperformed the conventional frequency-based method with an AUC of 0.740 (95% CI: 0.688–0.792, and *p* < 0.0001). Conclusions: Our findings indicate the potential of machine learning and transthoracic impedance measurements for estimating pleural effusion. By incorporating noninvasive and easily obtainable clinical and laboratory findings, this approach offers an effective means of assessing intrathoracic conditions.

## 1. Introduction

There are 64 million heart failure (HF) patients worldwide, including 1.2 million in Japan, and the number of people affected by it is increasing staggeringly [1,2]. It is the most common reason for hospitalization in people aged 65 and older, and has become a major economic burden [3,4]. The most common finding in this disease is pleural effusion, but chest examination requires chest X-ray or CT scan in a hospital. These specialized examinations are difficult for institutionalized and homebound older adults in terms of cost and mobility, contributing to delays in early detection and treatment. Thus, there is a need for a portable system that allows anyone to assess the condition of the thoracic cavity.

Pulmonary edema in patients with HF is a representative acute sign of congestion, and is defined as the increase in extravascular water volume [5]. The water content in the thorax can be monitored via an impedance method based on electrical resistance. Changes in intrathoracic impedance have been reported to be superior to changes in weight in predicting HF [6,7]. Current clinical methods of measuring impedance include implantable devices with pacemaker leads, but these can only be used in patients who have been implanted with such a device [6,7]. In contrast, a noninvasive diagnostic method is transcutaneous bioelectrical impedance analysis (BIA) [8,9]. Impedance values measured via BIA do not reflect just the condition in the thoracic cavity, but they are also strongly influenced by the general condition of the subjects, and the accuracy varies depending on the measurement conditions [8]. Consequently, although a few studies have investigated the use of impedance values to diagnose pulmonary edema, the diagnosis using absolute values is considered to be difficult [10,11].

On the other hand, a recent report suggested that a remote monitoring system that collects physical findings can be used to predict clinical worsening and the need for early therapeutic intervention [12]. In addition, studies have sought to apply machine learning to the prediction of cardiovascular disease [13,14]. We hypothesized that by constructing a prediction system using machine learning that incorporates impedance measurements, physical findings, blood test results, and weight changes, we might be able to predict the state of pleural effusion observed in patients with heart failure and its process with higher accuracy. In addition, the prediction system also learns about complications of pneumonia and infers the condition of the thoracic cavity.

The purpose of our study was to longitudinally collect various test results from the time of hospitalization to the discharge of the target patients and build a database. Furthermore, using this database, we aimed to develop a thoracic cavity estimation system that can be conveniently used by applying machine learning. Although we limited our discussion in this paper to the inference of the presence or absence of pleural effusion, we evaluated its predictive accuracy and aimed to build a technical foundation for the early detection of HF in the future.

## 2. Materials and Methods

### 2.1. Study Subjects

Between July 2021 and March 2022, patients diagnosed with HF or pneumonia and requiring hospital care were consecutively enrolled in this study. The control group consisted of healthy adult volunteers. The diagnosis was finalized by cardiologists based on congestive signs, symptoms, and laboratory findings. The patients with intra-thoracic treatment due to lung tumors, on a ventilator, or with electronic devices, such as cardiac pacemakers, were excluded. 

### 2.2. Data Collection

**Figure 1:**
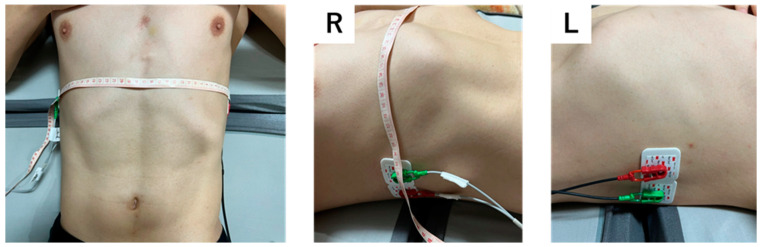
Electrode plate and attachment position. The left panel shows the picture in the front position. R and L indicate the right and left sides, respectively.

Hospitalized subjects were evaluated at least twice, at admission and before discharge if symptoms improved. In addition to impedance measurement, imaging studies, including chest CT and X-ray and blood tests, were performed at the time of admission, and chest X-ray and blood tests were performed before discharge. These tests were repeated depending on the clinical course of the hospitalized patients. Physical findings included height, weight and width. Physiological findings included blood oxygen saturation, blood pressure, and pulse. In addition to blood counts, the following were measured: renal function, liver function, and biochemical data. Impedance values were measured via a body composition analyzer (MLT-550N; SK Medical Electronics Co., LTD., Shiga, Japan) using the multi-frequency BIA (MFBIA) method. The MLT-550N is a BIA device that is commonly used in Japan. This device provides information on body composition using whole-body bioimpedance spectroscopy at 140 electrical frequencies via electrodes [15]. All BIA measurements were performed after at least 2 h of fasting. Four electrodes were used to measure BIA, two of which were placed side by side on the midline of each axilla and on the horizontal plane of the xiphoid process. The center of the electrode plate was placed at the intercostal position. Single-use electrocardiograph electrodes (3M™ Red Dot™ Foam Monitoring Electrode 2560, 3M Co., Red Wing, MN, USA) were used for the electrode plate (Figure 1). During BIA assessment, the subjects were in the supine position. The body analyzer measured body composition in terms of body water content, extracellular water volume (ECW), intracellular water content, body fat content, and body water fraction. The resistance (R) and reactance at 33 frequencies from 2.5 to 350 kHz, the impedance at infinite frequency (Z_inf_), the impedance at frequency 0 kHz (Z_0_), and the frequency at which the reactance was maximized (Fc; critical frequency) were measured. The phase angle (PhA) at each frequency and at Fc were also measured. PhA was calculated from the resistance (R) and capacitive reactance (Xc) using the following formula: arctangent (Xc/R) × 180°/π. These data were simultaneously accumulated from the MLT-550N, and the required time was about 4–5 min, including data entry for the subject and attachment of the electrode plate.

### 2.3. Machine Learning for Predictive Modeling

**Figure 2:**
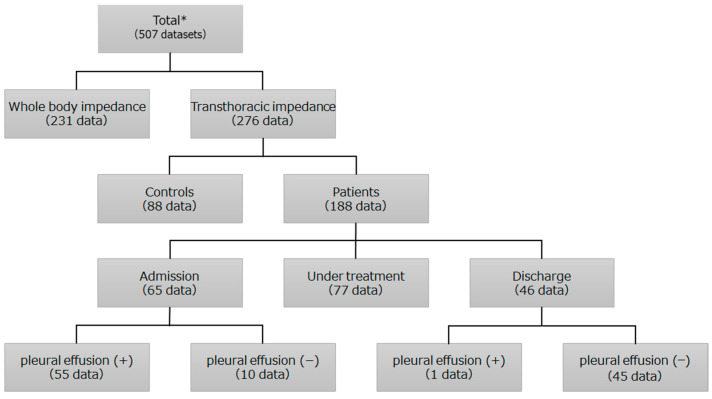
Background of the collected data. * including readmitted patients.

A total of 507 datasets were incorporated into the database, consisting of 63 patients with HF (including 7 patients with complications of pneumonia), and 82 healthy volunteers. These 507 datasets were included for database construction, with measurements taken from the bilateral axillae and from the right wrist and ankle for the same subject (Figure 2). Discrimination due to the measurement site was possible, and 276 datasets taken from both axillae were included for transthoracic impedance measurements. In total, 88 datasets were collected from 82 healthy volunteers, and 188 were collected from 63 patients during their hospital stay (Figure 2). During machine learning, it is possible to make judgments based on the measurement site. In this paper, we performed an evaluation based on transthoracic impedance measurements from both axillae. The constructed machine learning model uses data obtained by our measurement system as input items and performs binary classification of the presence or absence of pleural effusion. The machine learning model was implemented using the machine learning library light gradient boosting machine (LightGBM) and scikit-learn running on the Python programming language [16,17,18,19].

### 2.4. Preprocessing of Data

In data input to the machine learning model, missing value handling and feature generation were performed. For test items that are observed in the case of heart failure, we did not input the raw data. Instead, we used the binned features as the input values for machine learning. Weighting was performed based on the clinically obtained useful thresholds (Table 1), and each item was discretized in two or three stages to improve recognition accuracy and interpretability. For the thresholds of systolic blood pressure, we used a measurement of 160 mmHg, which is considered severe hypertension and is associated with a significantly increased risk of conditions, such as heart disease, stroke and kidney disease [20]. In addition, we used a measurement of 90 mmHg as the threshold for hypotension [21]. For heart rate, we referenced cases of atrial fibrillation commonly seen in heart failure patients, using 120 beats/min as the threshold for tachycardia and 40 beats/min as the threshold for significant bradycardia, where symptoms are likely to manifest [22,23]. For the C-reactive protein threshold, we used 10 mg/dL, which is considered to indicate a high likelihood of pneumonia, and 5 mg/dL, which indicates a moderate infection [24]. For those without clear thresholds, we set them based on the generally accepted normal range defined by past clinical experience. The missing data for each feature value were taken to be −1, a value that does not exist as a score. This was a method often used empirically as a replacement for features that can only take positive values. It allowed the machine learning model to explicitly learn that the value was missing. For electrical features affected by body size, we normalized them by using body width (BDW) and body surface area calculated using the Kurazumi formula [25]. We have defined body width as the horizontal distance between both the axillary midlines at the level of the xiphoid process. The impedance index (ZI_freq_) was normalized as the square of each subject’s BDW divided by the impedance at the appropriate frequency (Z_freq_), as in the following equation for impedance values:

ZI_freq_ = BDW^2^/Z_freq_ (cm^2^/Ω)

ZI_freq_ performs a logarithmic transformation to make the distribution closer to a normal distribution. In addition, for subjects whose cumulative data existed, the percentage of weight change from the time of the previous diagnosis was also generated as a feature value (Table 1).

### 2.5. Selection of Feature Values

Since the features including phase angle and impedance between the frequencies are highly correlated, the values at the specified frequencies and input features were selected using the following method. The filter method and wrapper method were used for feature selection to determine the factors related to pleural effusion. First, correlation coefficients between data were calculated, and items with strong correlations were deleted. Next, feature values with a relatively lower contribution to reasoning were deleted using LightGBM. Concerning other data, feature values that were clinically useful were first selected, and then features with relatively lower contributions were deleted using LightGBM in the same way. Table 1 shows a description of the feature value set for input to the machine learning model.

### 2.6. Development and Evaluation of Machine Learning Models

A four-fold cross-validation technique was used to validate the machine learning model. The machine learning model used LightGBM [19]. Two methods were used to evaluate our developed model. The first evaluation aimed to determine how useful the model was for predicting pleural effusion. This evaluation was performed by estimating HF using selected feature values as inputs. The second evaluation was conducted to clarify the electrical index (impedance) that was effective for predicting of pleural effusion. In this paper, we compared Z_0_, which is considered to be 0 kHz, with Z_50_, which has commonly been used in the past, and with our model. All impedance values are normalized based on the conversion equation described in “Preprocessing of data”. AUC, accuracy rate, sensitivity, and specificity were calculated to quantify the model’s performance. In addition, we ensured the reliability of our model by configuring it not to learn from the data of the same individuals that would be the subjects of inference during the implementation of cross-validation.

### 2.7. Analysis Methods

Measurement data are presented as the median and interquartile range (IQR) for variables with a non-normal distribution and as mean ± SD for those with a normal distribution. Measurement data were analyzed using the Mann–Whitney U test (non-normal distribution). Categorical data are expressed as n (%) and analyzed via the Chi-squared test. The DeLong test was used to compare the AUC between models. The tests were two-sided and *p* < 0.05 was considered to be statistically significant.

The data were processed using the packages numpy (version 1.14.3), pandas (version 0.23.0), and scikit-learn (version 0.19.1), which run on the Python (version 3.5.5) programming language. LightGBM (https://pypi.org/project/lightgbm/2.2.0/ (accessed on 1 July 2023), version 2.2.0) was used as the machine learning model. SHAP (https://pypi.org/project/shap/0.41.0/ (accessed on 1 July 2023), version 0.41.0) was used to interpret the models. SHAP is calculated based on the constructed LightGBM model and an index to explain the machine learning model based on game theory [26,27].

## 3. Results

### 3.1. Overview of the Estimation System

**Figure 3:**
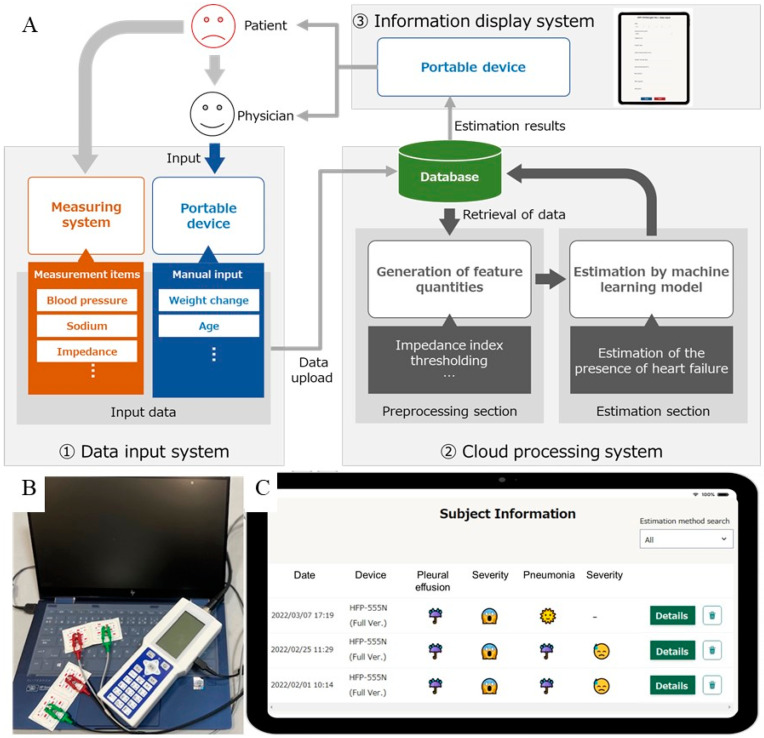
Configuration of the estimation system. (**A**) Overview of the estimation system, including (1) data input, (2) cloud processing, and (3) information display system. (**B**) HFP-555N. Data are sent from the device to a computer via USB. (**C**) Web-based user interface.

The estimation system consisted of (1) a data input system, (2) a cloud processing system, and (3) an information display system (Figure 3A). On the basis of the MLT-550N body composition analyzer, the HFP-555N analyzer with improved inputs of the items and outputs to the database was prepared (Figure 3B). The measured items are the same as those with the MLT-550N. The data input system allows the user to input data either manually or by uploading a file. It supports the use of tablets and smartphones in addition to personal computers. The cloud processing system stores the data entered in step (1) in a database in the cloud. The information display system allows the user to access the results on their devices (Figure 3C). The models uploaded to the cloud processing system were trained on all data in the datasets using the features and parameters described in the model prediction performance section.

### 3.2. Model Prediction Performance

**Figure 4:**
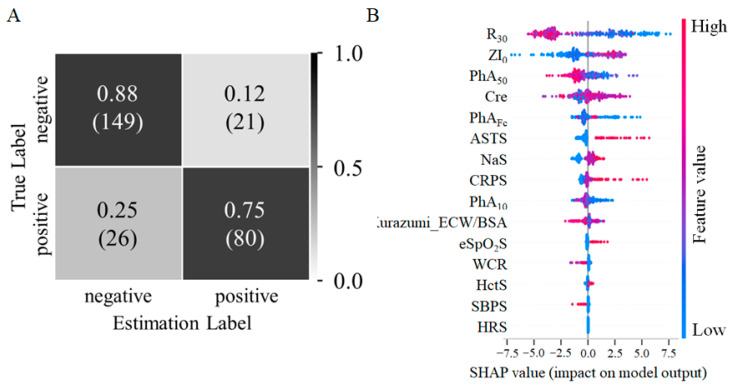
Accuracy and interpretation of pleural effusion inference model. (**A**) Confusion matrix in the model with LightGBM. The vertical axis indicates true labels, and the horizontal axis indicates predicted labels. (**B**) SHAP summary chart. The vertical axis shows the contribution to the predicted value, and the horizontal axis shows the positive or negative correlation of the feature value to the predicted value. The color of the feature value indicates the magnitude of its value in inferring pleural effusion. LightGBM = light gradient boosting machine; SHAP = Sapley additive explanation.

Figure 4A shows the confusion matrix with LightGBM. The accuracy of predicting pleural effusion was 0.830 (95% CI: 0.785–0.874), with a sensitivity of 0.755 (95% CI: 0.704–0.805) and specificity of 0.876 (95% CI: 0.838–0.915), using 276 transthoracic datasets. Figure 4B reflects the relationship between the feature value and the predicted probability through the use of color, including positive and negative prediction effects. Feature values with larger SHAP scores and stronger influences are located higher in the figure, and the results showed a more substantial influence of R_30_, ZI_0_, and PhA_50_. The color of the plot helped determine whether a feature value had a positive or negative relationship with the outcome. 

**Figure 5:**
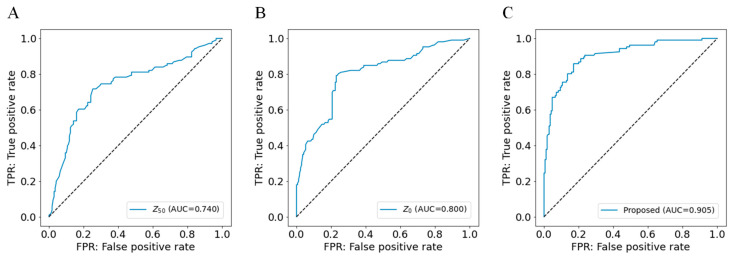
Receiver operating characteristic curves of three models. (**A**) Inference with Z_50_, (**B**) inference with Z_0_, (**C**) inference with our proposed features. AUC, the area under the curve.

The accuracy of our model was compared using only the impedance values Z_50_ and Z_0_. Figure 5A–C show the ROC curves and AUC of Z_50_, Z_0_, and our model. The AUC was 0.740 (95% CI: 0.688–0.792, and *p* < 0.001), 0.800 (95% CI: 0.753–0.847, and *p* < 0.001), and 0.905 (95% CI: 0.870–0.940, and *p* < 0.001), respectively. The AUC for Z_0_ was significantly larger than the conventional Z_50_ (*p* < 0.034), and our model had a significantly larger AUC than Z_0_ (*p* < 0.001). The AUC of our proposed model combining impedance with various features was significantly larger than the AUC of any model using impedance alone (*p* < 0.001).

### 3.3. Subject Characteristics

Two of the sixty-three patients required readmission within the study period. Table 2 shows the characteristics of the healthy controls and patients at discharge without pleural effusion (with improvement after treatment). The patient group had significantly older age, smaller body size, and a higher proportion of males. For electrical features, we observed significant differences in Z_inf_ and Z_0_. However, after normalization, as described in Section 2.4, these differences disappeared. No significant differences were found in the PhA at the critical frequency. Table 3 shows the subject characteristics regarding the presence or absence of pleural effusion in all transthoracic impedance datasets. The group with pleural effusion had a significantly higher proportion of males, were older, and had a smaller body stature. Regarding the electrical characteristics, all impedance values were significantly lower, and the PhA at the critical frequency also showed a lower value. The impedance index was also significantly larger than the group without pleural effusion. The discharge group that improved with treatment showed significant increases in each impedance value (decrease in the impedance index), reactance and phase angle.

## 4. Discussion

In this study, we devised a non-invasive, easy-to-use system for estimating intrathoracic conditions. This system, which employs machine learning, not only supports accurate diagnosis but also allows for continuous data collection in the future. By incorporating readily obtainable test values into our estimations, rather than relying solely on existing impedance values, we were able to achieve greater accuracy.

Bioelectrical impedance studies were reported from the 1950s to the 1980s, including by Beker et al. [28,29,30]. These studies led to the development of electrical impedance tomography (EIT) for quantitative evaluation of the respiratory system [31,32]. However, EIT still has the problem of intra- and inter-patient reproducibility, since the images obtained vary depending on the position of the electrodes, in addition to the large size of the device [32,33]. Some studies, most notably the SENSE-HF study, aimed at the early detection of HF from intrathoracic impedance [34]. In these studies, the measuring devices were implantable in the body and their effectiveness has been verified with applications to telemedicine. However, in addition to the fact that these approaches require an invasive procedure, many HF patients are not candidates for this implantable device [35,36].

Previous studies have described an edema guard monitor, with which measurements can be obtained from outside the body [37]. This device, with three electrodes attached to the right anterior chest and back, uses a special algorithm to determine intrathoracic impedance [38]. With this algorithm, intrathoracic impedance is calculated by subtracting the impedance on the skin surface from the transthoracic impedance required percutaneously. In this study, the method used to measure impedance was straightforward, with electrode plates attached to both axillae using the conventional percutaneous measurement method. As shown in Table 2 and Table 3, even with differences in age and body size, no differences were observed in impedance index and phase angle at critical frequency in the healthy state. Therefore, by normalizing with BDW, it was suggested that in normal health it may be possible to perceive any group as normal based on impedance measurements. On the other hand, during the presence of pleural effusion, each electrical feature showed a significant difference depending on whether pleural fluid was present or not. This suggests that this study has demonstrated the potential for inferring pleural effusion using electrical features, including impedance index. Unsurprisingly, the previously noted issues with reproducibility remain. However, this method may at least be useful as a clinical tool for assessing pulmonary effusion [39,40,41]. The results of this study indicated that even by using existing measurement device, machine learning can provide higher accuracy in inference than conventional methods. As the amount of data regarding electrical characteristics, physiological and physical findings, and hematological findings at the measurement time increased, the predictive accuracy improved. However, intrathoracic impedance and simple physiological findings alone were sufficiently predictive.

One of the key points in this study was how to evaluate the measured impedance and resistance with respect to individual body size and gender differences. Okazaki et al. reported that gender and body size, but not age, effected impedance [40]. Therefore, we first normalized the impedance and resistance values by body width and incorporated these values into the evaluation equation, and then normalized the ECW values using body surface area. For body surface area, we used the formula described by Kurazumi et al., which reflects the gender and body shape of the Japanese people [25,42].

Next, we discuss the selection of the frequencies and other electrical parameters used in our model. Multiple frequencies in MFBIA have been reported to provide a more accurate assessment of intracellular and extracellular fluids than single-frequency alternating current BIA [8,43,44]. Specifically, in our model, we ultimately selected impedance values at 0 kHz and 30 kHz in addition to a frequency of 50 kHz, which is commonly used in the BIA method. It was also reported that PhA, one of the parameters of BIA, was positively correlated with plasma membrane integrity; PhA decreased when the plasma membrane was damaged and membrane function was reduced [45]. A lower PhA indicated the presence of nutritional disorders and may reflect body fluid imbalances, such as edema, due to acute inflammation or hypoalbuminemia [46]. In HF and pneumonitis, the permeability of the plasma membrane is thought to be elevated by osmotic pressure or inflammation. As shown in Table 3, the PhA at the critical frequency and at 50 kHz significantly decreased during pleural effusion, which supported the previous report. Therefore, we used PhA as a parameter. Regarding the frequency used for PhA, most previous reports have used 50 kHz, which is considered to provide the largest reactance [8,47]. From a phase angle of 2–250 kHz, we selected 10 kHz, 50 kHz, and the phase angle at the critical frequency at which the reactance is maximized in diseased conditions.

Physical findings, such as tachycardia, abnormal blood pressure, weight gain, tachypnea, and blood tests, such as renal function, electrolytes, and complete blood, count are also important for diagnosis [48]. In this study, we have built a data collection system and a database that includes blood test results, physiological findings, and personal historical data that allow comparison with past conditions. Furthermore, using this database, we have built a machine learning model with features that include data binned according to the threshold based on previous research and clinical findings (as shown in Section 2.4, Data Preprocessing), and we confirmed its effectiveness.

Finally, all of the electrical parameters are automatically obtained from the instrument. In addition, each of the other parameters has been turned into an algorithm that allows estimation regardless of the presence or absence of some measurement inputs. The algorithm has been developed based on actual clinical practice, assuming that use of the system and the amount of information available are different in private homes, nursing homes, clinics, and hospitals. Our results were close to those in Figure 5A,B when no parameters other than electrical parameters were available, and as the number of parameters increased, the results became closer to those in Figure 5C. In clinical practice, physical and physiological findings are also essential for the diagnosis of HF [49]. In addition, these examination findings can be measured in nearly all situations, even when blood tests cannot be conducted, making their use as variables for estimation reasonable.

Compared to traditional risk estimation methods, the strength of machine learning is its ability to detect complex nonlinear relationships and iteratively improve its models with more data, resulting in more accurate estimates and fewer false alarms [14,50]. Examples of predictive models enhanced by machine learning include those that predict arrhythmia, cardiac arrest, and thromboembolism [51]. They are expected to be applied to preventive medicine [52]. 

As illustrated in Figure 3C, we have developed a machine learning-based intrathoracic estimation system on the cloud, featuring a web user interface that is easy for anyone to use. This system provides useful information for disease determination and simultaneously allows for the collection of data that could potentially enhance the accuracy of disease identification in the future. The ongoing accumulation of data paves the way for the creation of a high-precision diagnostic model that can consider multiple diseases, based on non-invasive collection of impedance values.

This study had some limitations. First, this study was initiated based on our previous research in which we observed changes in impedance values during pleural effusion in pigs, and it aimed to evaluate the presence or absence of pleural effusion. HF is classified into pulmonary congestion, pulmonary edema, and the more advanced condition of pleural effusion. It is necessary for future research to aim at evaluations at earlier stages before the onset of pleural effusion. In this regard, few patients in the present study had only mild congestion. To make predictions about the early stages of heart failure or very mild heart failure, we need more patient data about pulmonary congestion and pulmonary edema. Second, we could not recruit age-matched healthy volunteers at the time of the study due to the impact of a pandemic caused by the SARS-CoV-2 virus, and the control data were collected from healthy people in a relatively younger age group who had a low risk of morbidity. In Table 2, it can be seen that even where there is a difference in age or BMI, our impedance index value did not show a significant difference during normal health. However, we cannot completely rule out the possibility that factors associated with this collection method may have influenced the accuracy of our inference model. Third, regarding the electrical characteristics in the test results, several cases were found with F_c_ and PhA values of 0 kHz and 0 degrees, respectively. The reason for this should be investigated in the future. Fourth, our estimation system must be validated in subjects at various pre-diagnostic stages and in healthy subjects. As we move forward with new research, it will be necessary to conduct power analysis and tests of independence to ensure the reliability and generality of our model, thereby strengthening our claims. On the other hand, the fact that many older patients have pleural effusions when they are admitted to the hospital suggests that our system may be useful in clinical practice.

## 5. Conclusions

The system developed in this study has demonstrated the capability to estimate intrathoracic fluid retention with greater accuracy than conventional methods by using transthoracic impedance and other measurements, thereby suggesting its clinical utility. Our system allows for concurrent data collection during estimation. Ultimately, this could enable the development of a highly accurate intrathoracic estimation model based on impedance values using the large datasets we have collected. We have already developed a system to display the severity of pleural effusion; however, a larger sample size is essential for more accurate evaluations, presenting a challenge for future work. We aim to develop a reliable sensitivity algorithm for this approach.

## Figures and Tables

**Table 1:** Feature values used in machine learning.

Variables	Score	Feature Value
SBP (mmHg)	160:1	SBPS
	90–160:0	
	<90:1	
HR (bpm)	>120:1	HRS
	40–120:0	
	<40:1	
eSpO_2_ (%)	≥90:0 <90:1	eSpO_2_S
Hct (%)	>50.0:1	HctS
	30.0–50.0:0	
	<30.0:1	
AST (U/L)	>40:1	ASTS
	10–40:0	
	<10:1	
Na (mEq/L)	>145:1	NaS
	135–145:0	
	<135:1	
CRP (mg/dL)	<5.0:0 5.0–10.0:1 ≥10.0:2	CRPS
Weight change percentage from a previous examination (%)	N/A	WCR
PhA at 10 kHz (degrees)	N/A	PhA_10_
PhA at 50 kHz (degrees)	N/A	PhA_50_
PhA at critical frequency (degrees)	N/A	PhA_Fc_
R at 30 kHz (ohm)	N/A	R_30_
ZI at 0 kHz (cm^2^/ohm)	N/A	ZI_0_
ECW normalized via BSA * (kg/m^2^)	N/A	Kurazumi_ECW/BSA

SBP, systolic blood pressure; HR, heart rate; eSpO_2_, estimated SpO_2_; Hct, hematocrit; Alb, albumin; AST, aspartate aminotransferase; CRP, C-reactive protein; PhA, phase angle; R, resistanceimpedance; ZI, impedance index; ECW, extracellular water; BSA, body surface area * Kurazumi’s formula for males: BSA = 53.189 W^0.326^ H^0.833^ × 10^−4^; Kurazumi’s formula for females: BSA = 110.529 W^0.445^ H^0.627^ × 10^−4^; W, weight (kg); H, height (cm).

**Table 2:** Characteristics of healthy controls and patients at discharge without pleural effusion.

Variable	Healthy Controls (88 Datasets)	Patients at Discharge Without Pleural Effusion (45 Datasets)	*p*-Value
Gender (Male) (%)	27.3	46.7	0.025
Age (years)	44.8 ± 13.2	84.3 ± 11.1	<0.001
Body weight (kg)	60.5 ± 13.2	45.0 ± 13.8	<0.001
Height (cm)	161.1 ± 7.9	153.1 ± 10.7	<0.001
BMI	23.2 ± 4.0	18.9 ± 4.2	<0.001
Body width (cm)	32.0 ± 4.0	27.7 ± 3.3	<0.001
Z_inf_ (Ω)	41.3 ± 16.0	31.6 ± 12.0	<0.001
Z_0_ (Ω)	67.2 ± 17.2	50.9 ± 20.4	<0.001
F_c_ (kHz)	61.4 ± 23.9	59.1 ± 74.2	0.132
PhA_Fc_ (degrees)	8.5 ± 2.4	11.7 ± 11.9	0.127
PhA_50_ (degrees)	8.2 ± 2.4	5.2 ± 3.1	<0.001
ZI_inf_ (cm^2^/ohm) *	27.3 ± 8.2	27.8 ± 12.7	0.866
ZI_0_ (cm^2^/ohm) *	15.9 ± 3.7	17.1 ± 8.6	0.529

BMI, body mass index; Z_inf_, impedance at infinite Hz; Z_0_, impedance at 0 kHz; F_c_, critical frequency; PhA_Fc_, phase angle at critical frequency; PhA_50_, phase angle at 50 kHz; ZI_inf_, impedance index at infinite Hz; ZI_0_, impedance index at 0 Hz * ZI_freq_ = (body width)^2^/Z_freq_; ZI_freq_, impedance index at the appropriate frequency; Z_freq_, impedance at the appropriate frequency.

**Table 3:** Characteristics of subjects with and without pleural effusion.

Variable	Pleural Effusion + (106 Datasets)	Pleural Effusion − (170 Datasets)	*p*-Value
Gender (Male) (%)	49.1	34.7	0.018
Age (years)	86.3 ± 9.1	63.9 ± 23.0	<0.001
Body weight (kg)	47.7 ± 13.7	52.6 ± 15.3	0.009
Height (cm)	151.6 ± 10.3	157.3 ± 10.1	<0.001
BMI	20.7 ± 4.6	20.9 ± 4.6	0.632
Body width (cm)	28.2 ± 3.4	29.8 ± 4.4	0.002
Z_inf_ (Ω)	22.6 ± 9.7	35.6 ± 15.0	<0.001
Z_0_ (Ω)	34.8 ± 34.2	56.7 ± 20.9	<0.001
F_c_ (kHz)	67.4 ± 71.2	57.1 ± 45.9	0.644
PhA_Fc_ (degrees)	8.4 ± 11.5	9.5 ± 7.6	<0.001
PhA_50_ (degrees)	3.9 ± 2.1	6.8 ± 2.9	<0.001
ZI_inf_ (cm^2^/ohm) *	43.2 ± 28.7	28.2 ± 11.0	<0.001
ZI_0_ (cm^2^/ohm) *	23.3 ± 13.1	17.4 ± 7.4	<0.001

BMI, body mass index; Z_inf_, impedance at infinite Hz; Z_0_, impedance at 0 kHz; F_c_, critical frequency; PhA_Fc_, phase angle at critical frequency; PhA_50_, phase angle at 50 kHz; ZI_inf_, impedance index at infinite Hz; ZI_0_, impedance index at 0 Hz * ZI_freq_ = (body width)^2^/Z_freq_; ZI_freq_, impedance index at the appropriate frequency; Z_freq_, impedance at the appropriate frequency.

## Data Availability

The de-identified participant data will not be shared.
